# Combined Cisplatinum and Laser Thermal Therapy for Palliation of Recurrent Head and Neck Tumors

**DOI:** 10.1155/DTE.6.133

**Published:** 2000

**Authors:** Marcos B. Paiva, Romaine E. Saxton, Keith E. Blackwell, Peter Buechler, Alen Cohen, Carson D. Liu, Thomas C. Calcaterra, Paul H. Ward, Dan J. Castro

**Affiliations:** 1 Division of Head and Neck Surgery UCLA School of Medicine 62-132 CHS, 10833 Le Conte Avenue Los Angeles CA 90024-1624 USA; 2 Division of Surgical Oncology UCLA School of Medicine 31-24 Rehab Building 1000 Veteran Ave. Los Angeles CA 90095-1794 USA; 3 Division of General Surgery UCLA School of Medicine 31-24 Rehab Building 1000 Veteran Ave. Los Angeles CA 90095-1794 USA

## Abstract

In recent years endoscopically controlled laser-induced thermal therapy (LITT) has been increasingly accepted as a minimally invasive method for palliation of advanced or recurrent head and neck or gastrointestinal cancer. Previous studies have shown that adjuvant chemotherapy can potentiate endoscopic laser thermal ablation of obstructing tumors leading to improved palliation in advanced cancer patients. Eight patients with recurrent head and neck tumors volunteered to enroll as part of an ongoing phase II LITT clinical trial, and also elected to be treated with systemic chemotherapy (cisplatin, 80 mg/m^2^) followed 24 h later by palliative laser thermal ablation. Laser treatments were repeated in patients with residual disease or recurrence for a total of 27 LITT sessions. Four of the 8 patients treated with laser thermal chemotherapy remained alive after a median follow-up of 12 months. Of the 12 tumor sites treated, complete responses were located in the oral cavity (3), oropharynx (1), hypopharynx (1), maxillary sinus (1), and median survival for these patients was 9.5 months. This initial experience with cisplatinum-based laser chemotherapy indicates both safety and therapeutic potential for palliation of advanced head and neck cancer but this must be confirmed by longer follow-up in a larger cohort of patients.


*Diagnostic and Therapeutic Endoscopy*, Vol. 6, pp. 133–140
Reprints available directly from the publisher
Photocopying permitted by license only

© 2000 OPA (Overseas Publishers Association) N.V.
Published by license under
the Harwood Academic Publishers imprint,
part of The Gordon and Breach Publishing Group.
Printed in Malaysia.

### Combined Cisplatinum and Laser Thermal Therapy for Palliation of Recurrent Head and Neck Tumors

MARCOS B. PAIVA[a], ROMAINE E. SAXTON[b], KEITH E. BLACKWELL[a], PETER BUECHLER[c], ALEN COHEN[a], CARSON D. LIU[c], THOMAS C. CALCATERRA[a], PAUL H. WARD[a] and DAN J. CASTRO[a,*]

[a]*Divisions of Head and Neck Surgery,* [b]*Surgical Oncology,* [c]*General Surgery, UCLA School of Medicine, 62-132 CHS, 10833 Le Conte Avenue, Los Angeles, CA 90024-1624, USA; 31-24 Rehab Building, 1000 Veteran Ave., Los Angeles, CA 90095-1794, USA*

*(Received 16 August 1999; Revised 18 October 1999; In final form 17 November 1999)*

**In recent years endoscopically controlled laser-induced thermal therapy (LITT) has been increasingly accepted as a minimally invasive method for palliation of advanced or recurrent head and neck or gastrointestinal cancer. Previous studies have shown that adjuvant chemotherapy can potentiate endoscopic laser thermal ablation of obstructing tumors leading to improved palliation in advanced cancer patients. Eight patients with recurrent head and neck tumors volunteered to enroll as part of an ongoing phase II LITT clinical trial, and also elected to be treated with systemic chemotherapy (cisplatin, 80 mg/m$^2$) followed 24 h later by palliative laser thermal ablation. Laser treatments were repeated in patients with residual disease or recurrence for a total of 27 LITT sessions. Four of the 8 patients treated with laser thermal chemotherapy remained alive after a median follow-up of 12 months. Of the 12 tumor sites treated, complete responses were located in the oral cavity (3), oropharynx (1), hypopharynx (1), maxillary sinus (1), and median survival for these patients was 9.5 months. This initial experience with cisplatinum-based laser chemotherapy indicates both safety and therapeutic potential for palliation of advanced head and neck cancer but this must be confirmed by longer follow-up in a larger cohort of patients.**

*Keywords:* Chemotherapy, Cisplatin, Head and neck cancer, Laser-induced thermo-therapy, Nd : YAG laser, Squamous cell carcinoma

### INTRODUCTION

Laser induced thermal therapy (LITT) using the Nd:YAG laser via endoscopic fiberoptics has been increasingly accepted as an effective palliative method for tumor ablation [1–4]. LITT facilitated by endoscopy is of great value for less invasive palliation of advanced obstructing cancers of the head and neck, as well as for the gastrointestinal and bronchial tracts by endoscopic recanalization [1,2,4]. High photo-thermal laser energy levels are delivered to the area of maximum obstruction but

* Corresponding author. Tel.: +1 310 825 8954. Fax: +1 310 206 9840. E-mail: mpaiva@mednet.ucla.edu.

lower levels to the tumor margins, which frequently remain undertreated resulting in recurrences [1–4]. Despite well documented tumor responses and an apparently increased patient survival after LITT, the need for repeated palliative laser sessions suggests that more effective treatment may be possible by combined drug and laser therapy [5–11]. Cisplatin in combination with other anticancer agents has been successfully used together with Nd : YAG laser therapy for endoscopic palliative treatment of advanced esophageal and gastric cancer [6–11] and is a current clinical protocol as an alternative to radiation therapy for treatment of retinoblastomas as reported by several investigators [12–14]. The rationale for combined laser and chemotherapy is based on the observation that tumor recurrence after LITT occurs at the margin and that drug enhancement by hyperthermia at these sites could eliminate or reduce this tumor regrowth [7,8].

### PATIENTS AND METHODS

Based on reports that chemotherapy enhances palliative endoscopic laser therapy in advanced esophageal and gastric cases [6–11], the authors began a pilot study from July 1994 to July 1998, testing combined cisplatin and laser induced thermal therapy in 8 patients with recurrent head and neck tumors who were not candidates for conventional surgery and radiation treatment. Treatments were not limited to endoscopic laser ablation of recurrent tumors in the oropharynx, larynx and hypopharynx, but also included other tumor sites such as the oral cavity, maxillary sinus, neck and skin. The treatment schedule was divided into two phases. An initial treatment phase occurred when patients were administered cisplatin followed by laser treatment with the intention of relieving symptoms via drug enhanced ablation of recurrent tumor. The follow-up phase occurred when the patients returned for maintenance laser therapy in cases where post-operative tumor regrowth was visible or microscopic biopsies identified positive tumor margins.

For initial treatment each patient was admitted on the day before surgery and administered cisplatin, 80 mg/m$^2$ intravenously over 1 h and scheduled for LITT tumor ablation the next day. All subjects were examined by flexible fibroscopy and radiological exam to assist in adequate tumor staging and to evaluate spread of disease. These patients were treated by Nd : YAG laser ablation as part of a Phase II study that evolved in a step-wise fashion over the last decade [15]. Patients were excluded if the Karnofsky performance status was less than 70% or in cases with tumor immediately adjacent to the carotid artery, esophagus or trachea. Tumor volume, was measured using the formula = $(L \times W \times H)/2$, where $L$ is the greatest diameter, $W$ is the greatest perpendicular width, and $H$ is the greatest depth or height. Tumor volume ranged from 1.5 to 105 cm$^3$. Patient's medical records and follow-up were available to accurately assess treatment area, tumor size, and outcome in recurrent and/or metastatic unresectable tumors that were unresponsive to previous treatment. Age distribution of the reviewed cases ranged from 50 to 85 years, with a peak incidence in the seventh decade. The 6 men and 2 women each presented with one or 2 tumors and all had a past history of cigarette smoking. All patients had a diagnosis of squamous cell carcinoma confirmed by biopsies.

All LITT procedures were performed in the operating room, usually on an outpatient basis. After obtaining appropriate informed consent, the patients were placed on continuous cardiac and pulse oximetry monitoring and then heavily sedated in 37% of cases, or placed under general anesthesia (63%). Initially, biopsies were performed at the tumor margins using a clockwise orientation before starting laser therapy. An SLT Nd : YAG laser (1064 nm, SLT Co. Oaks, PA) was employed for all tumor treatments, with the intention to thermally ablate tumor tissue. The free beam laser energy at continuous mode (50 W) was delivered via fiberoptics for a total of 11 endoscopic procedures, or through a handpiece in 16 procedures. Endoscopic procedures were performed in 3 patients by suspension microlaryngoscopy for ablation of tumors in the larynx, pharynx and oropharynx. The laser application was performed initially at 5 mm beyond the tumor margins in a centripetal configuration directing the light beam perpendicularly from above with the tip of the handpiece 5 mm over the target tissue. The beam was maintained for 5–7 s on each target spot to achieve an energy density of 1600 to 2200 J/cm$^2$ per application. After carbonization of the tumor, debris was removed by suction. Following completion of debulking, the tumor bed with margins of 10 mm was treated again with the laser in the same fashion using a similar energy density. This produced a deeper layer of photoablation and better tumor eradication as well as photocoagulation of smaller vessels.

All patients with recurrent neck tumors needed ultrasound assistance (UTZ) to facilitate localization of the tumor in relation to the carotid artery and ensure that laser energy deposition remained away from vital structures, as described previously [15]. After treatment all patients were followed by repeat visits to the outpatient clinic every 3–5 weeks where they were examined clinically and performance status was reassessed and recorded. Each patient's overall outcome after therapy was recorded as complete, partial or no response, based on duration of follow-up. Complete response (CR) was defined as no visible tumor; partial response (PR) exhibited a decreased initial tumor volume of > 50% followed by delayed regrowth; no response (NR) was defined by greater than 50% of pre-therapy tumor size or rapid tumor regrowth after treatment.

### RESULTS

Twelve recurrent tumors in 8 patients were treated by combined laser and cisplatin therapy, with tumors occurring in the oral cavity (3), skin (2), neck (2), oropharynx, hypopharynx, larynx, nose, and maxillary sinus. Overall, pain was the major complaint (4 patients), while other symptoms included dysphagia, dyspnea, bleeding, and difficulties in chewing. Table I gives a summary of the patients' profile. Twenty-seven laser procedures were performed: 8 were initial treatment phase using cisplatin as a booster for laser therapy and 19 were repeated procedures during follow-up phase. The mean energy required to produce remission was 22.5 kJ (range 4.8–54 kJ) in the initial treatment phase, and 9.3 kJ (range 3.6–21.5 kJ) in the follow-up phase. Average time interval between treatments was 4.8 weeks. Twenty of 27 LITT procedures were performed as a same-day surgery (74%) and 7 (26%) required one day hospitalization. Average anesthesia time for these procedures was 63 min which illustrates the speed and simplicity of combined LITT as an alternative to surgical resection. Laser and cisplatin treatments led to CR in 6 of 12 tumors treated. Five patients had improvement and/or resolution of symptoms after one to 6 sessions (average of 3.3 procedures per patient). Detailed results of these treatments are included in Table II.

A 52-year-old male (patient #2) was unresponsive to conventional treatment. He presented with

TABLE I Clinical profile of patients selected for Nd : YAG laser and cisplatin combined therapy

| Patient no. | Sex/age | Previous treatment | Reason for treatment | SCCA Histol. differentiation | Treatment site | Tumors (*n*) | Tumor vol. range cm$^3$ |
|---|---|---|---|---|---|---|---|
| 1 | m/60 | Surg/rad/chemo | Unresectable | Poor | Oropharynx | 1 | 50 |
| 2 | m/52 | Surg/rad/chemo | Unresectable | Poor | Nasal/oral cav/skin | 4 | 1.5–3.8 |
| 3 | m/60 | Rad/chemo | Refused surg/rad | Poor | Floor of Mouth | 1 | 32 |
| 4 | m/69 | Rad | Refused surg/rad | Moderate | Larynx | 1 | 5.4 |
| 5 | f/85 | Surg/rad/chemo | Not surg. candidate | Moderate | Hypopharynx | 1 | 2.4 |
| 6 | m/50 | Surg/rad/chemo | Unresectable | Poor | Oral Cavity/neck | 2 | 3.2–24 |
| 7 | m/70 | Surg/rad/chemo | Unresectable | Poor | Neck | 1 | 105 |
| 8 | f/75 | Surg/rad/chemo | Not surg. candidate | Poor | Maxillary sinus | 1 | 5.1 |

TABLE II Outcome and follow-up after Nd:YAG laser treatment

| Patient no. | Nd:YAG sessions | Sessions for negative biopsy | Survival (months) | Tumor metastasis | Status |
|---|---|---|---|---|---|
| 1 | 2 | 1 | 5.5 | no | Expired: (N.E.D.) |
| 2 | 4 | Remained pos. | 4 | no | Expired with disease |
| 3 | 2 | 1 | 56 | no | Alive in remission |
| 4 | 6 | 4 | 14 | no | Alive with disease |
| 5 | 3 | 3 | 10 | no | Alive in remission |
| 6 | 4 | Remained pos. | 5 | no | Expired with disease |
| 7 | 3 | Remained pos. | 8 | Lung | Expired with disease |
| 8 | 3 | 3 | 11 | no | Alive in remission |

recurrent rapidly growing facial subcutaneous (2 tumors), palatal and posterior septal poorly differentiated squamous cell tumors. Tumor ablation was achieved after initial combined therapy, but rapid regrowth occurred after every laser application and further treatment was stopped after 4 sessions. Patients #6 and #7 had reasonable palliation with some reduction in tumor growth rate but eventually expired due to further progression of the disease (5 and 8 months survival respectively). Targeting neck tumors with the external laser technique was difficult in these 2 patients with advanced neck disease due to deep tumor invasion around nervous plexi and vessels. Both patients had an initial combined cisplatin and LITT ablation under ultrasound (UTZ) guidance and a second LITT without cisplatin, also under UTZ guidance during the follow-up phase. Treatment of neck lesions was challenging because these tumors frequently impinge on vital structures after recurrence following failure of wide surgical resection. Patient #7 had extensive recurrence in the neck that encroached around the right common carotid artery with a partial response to initial laser and cisplatin therapy followed by 2 LITT sessions without chemotherapy, as seen in Fig. 1.

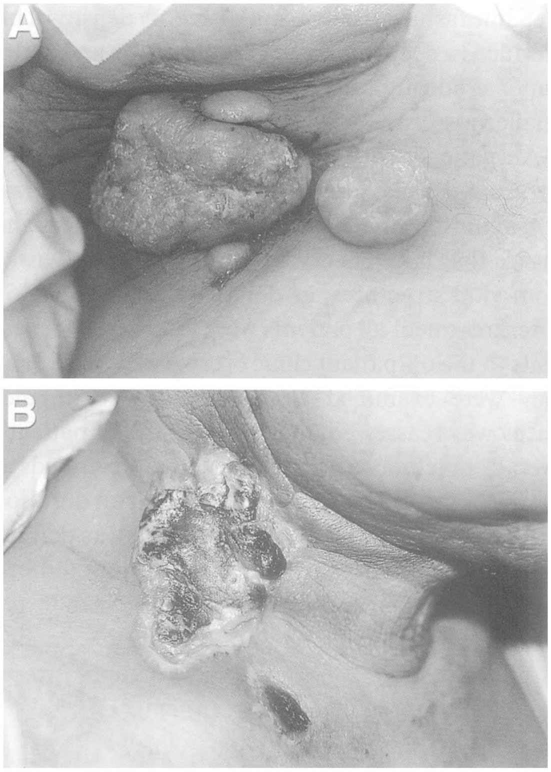

FIGURE 1 (A) Preoperative view of a right neck metastatic squamous cell cancer (patient #7), and (B) view 2 weeks after third treatment with the Nd:YAG laser showing a PR to treatment.

Four patients showed CR after combined treatment. Patient #1, a 60-year-old male presented with a rapidly growing left lateral pharyngeal wall and base of tongue poorly differentiated squamous cell carcinoma (50 cm$^3$) that failed previous surgery and radiation. The patient showed a dramatic tumor reduction and negative repeat biopsies after first laser and cisplatin treatment. Unfortunately, the patient had a stroke 5.5 months after initial treatment and could not be followed for long term response. Another patient (#4) also was not available for extended follow-up because he elected to discontinue further treatment. This 69-year-old male presented with a recurrent T4 laryngeal carcinoma, which had failed previous radiation therapy. He refused a total laryngectomy, but agreed to undergo cisplatin and LITT therapy via laryngoscopy for treatment of the carcinoma. This patient underwent other 5 LITT treatments, with negative tumor biopsies after 4 treatments. Fourteen months after first treatment, the patient had poor voice function and was breathing without a tracheotomy, but refused to undergo routine serial sessions of suspension laser microlaryngoscopy for biopsies during the follow-up phase of the study. The patient subsequently was reported to have undergone a total laryngectomy. Patient #3 who presented with a T4N0M0 squamous cell carcinoma of the floor of mouth had an apparent curative response after a single session of combined laser and cisplatin therapy with no evidence of tumor recurrence during 4 years follow-up (Fig. 2). Biopsies performed at 2.5 years after initial treatment were negative and this patient remains active with a good quality of life.

Minor side-effects of the initial treatment phase with cisplatin and laser were common to most of the patients including a sensation of weakness with no objective findings in 4 patients and nausea with vomiting in 2 patients. These symptoms spontaneously resolved within a few days except in patient # 8 who responded with mild alopecia (grade 2 NIH toxicity score). No intra-operative adverse medical reactions were seen when Nd : YAG laser LITT was administered after systemic cisplatin administration. This combined treatment was well tolerated in 7 patients at the selected laser power and drug levels, causing no discernible adverse effects to overlaying soft tissue or adjacent structures. As a palliative form of therapy, most laser treatments were performed on an outpatient basis (74%) and median survival in this series of patients was 9.5 months. The survival curve for the entire study is shown in Fig. 3.

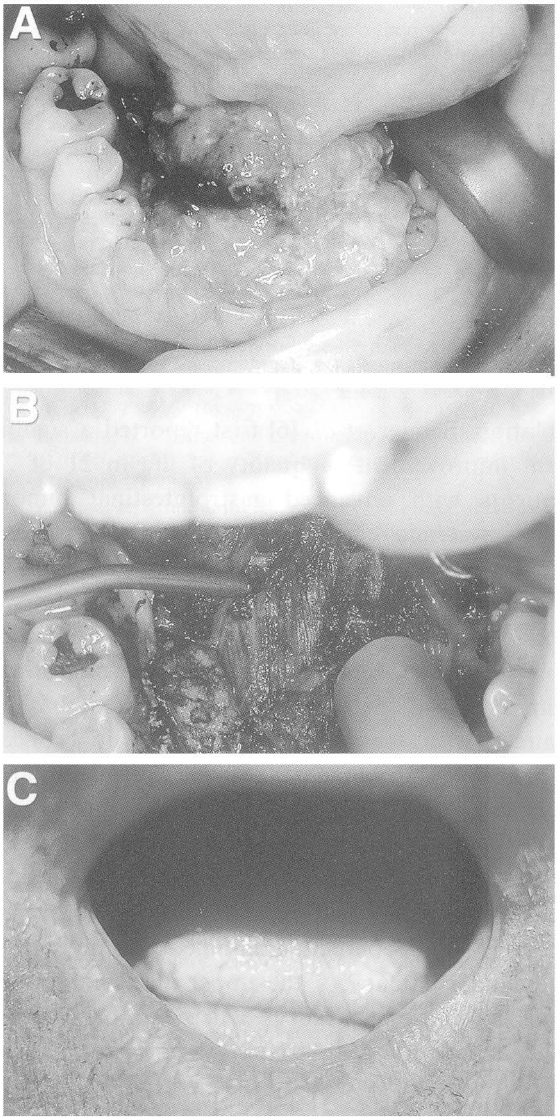

FIGURE 2 (A) Preoperative view of an extensive squamous cell cancer of the floor of mouth (patient #3), and (B) view immediately after treatment with cisplatin and Nd : YAG laser; and (C) post-operative result after 3 years, showing a well healed defect covered by a skin flap.

### DISCUSSION

Local recurrence after endoscopic laser thermal palliation is frequently related to residual disease from untreated areas in the tumor margins. Repeated laser thermal ablation sessions are usually needed for effective palliation to induce eventually tumor remission using LITT. In an effort to improve palliative endoscopic laser thermal ablation of advanced malignancies, several investigators have tested combined chemotherapy prior to tumor

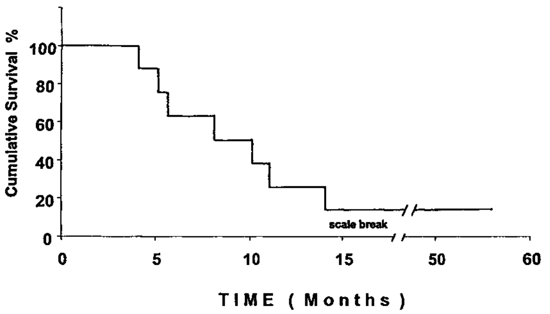

FIGURE 3 Kaplan Meier survival curve of patients measured in months after the initial treatment.

ablation. Semler *et al.* [6] first reported a significant improvement of quality of life in 21 of 24 patients with advanced gastrointestinal tumors when systemic chemotherapy was administered before intraluminal endoscopic Nd:YAG laser thermal ablation. Mache [7] confirmed survival benefits of this alternative combined treatment for palliation of advanced upper gastrointestinal tumors. Firusian [8] previously reported that 13 patients with stenotic upper gastrointestinal cancer treated by endoscopic palliative laser ablation and cisplatin in combination with other anticancer drugs, responded favorably after combined laser chemotherapy compared to laser alone as a palliative approach for advanced malignant disease. In most of the 13 patients combined therapy led to immediate patency of the upper alimentary tract and 8 months median survival in the cohort studied. The author also found that local laser thermal effects were enhanced by systemic chemotherapy leading to additional ablation of malignant cells down to 7–8 mm depth compared to 4 mm for laser alone treatment. Combined drug and laser therapy was more effective in preventing rapid cell proliferation at the tumor margins [8].

Cisplatin combined with other chemotherapy agents in different regimens has been the drug of choice in several laser endoscopic palliative studies [9–11]. Mason [11] reported a study on endoscopic Nd:YAG laser ablation comparing treatment alone versus laser plus systemic chemotherapy in patients with inoperable esophageal cancer and observed that combined therapy produced a response in 'two-thirds of cases with relief of dysphagia and increase in survival. The author also compared 2 groups receiving combined laser and chemotherapy treatments FA/5-FU (Folinic acid and Fluoracil, i.v.), and ECF (Epirubicin, cisplatin and 5-Fluoracil, i.v.). No response was seen in patients treated with FA/5-FU, compared to a 61% response in patients treated by ECF with a significant reduction in need for additional laser therapy to maintain swallowing [11].

Accumulating evidence indicates that heat induction causes ultrastructural changes in tumor cell membranes leading to increased membrane transport of drugs and altered cellular metabolism [16,17]. Higher CDDP uptake after heating also enhances cytotoxicity in proliferating cancer cells and stimulates protein kinases to induce tumor apoptosis [18]. Studies of CDDP binding to DNA have shown that hyperthermia induces more DNA adducts, contributes to increased tumor growth delay, and amplifies interstrand crosslinks by cisplatinum [19–23]. Together, these results provide strong evidence that synergistic effects of heat and cisplatinum are likely to improve therapeutic response of recurrent head and neck tumors following combined drug and laser treatment.

A drug implant of cisplatinum with collagen as a protein matrix and epinephrine, (CDDP/gel, or Intradose™) allows higher intratumor drug levels to be maintained for nearly 24 h [24]. This slow release drug gel has been found to be safe and feasible for palliation of esophageal as well as head and neck and liver tumors [24]. The CDDP drug implant also may provide an alternative combined technique with LITT for more effective therapy without systemic drug toxicity. Combining CDDP/gel with LITT in a human squamous cell carcinoma tumor transplant model resulted in significant therapeutic improvement compared to systemic or intratumor injection of cisplatin in several recent pre-clinical studies [5,25,26]. Currently, patients with advanced head and neck cancer are palliated at UCLA either by laser ablation in a phase II study or by intralesional cisplatin injection (CDDP/epi-gel, phase III study) with clear evidence of tumor regression in a number of patients, but with eventual recurrence in the majority of cases [15,24].

An obvious next step is to combine intratumor cisplatin injections with interstitial Nd : YAG laser therapy for cancer treatment [5,26].

The options for active palliation of local symptoms in inoperable patients are usually very limited [2,15]. Although the current study was based on a small number of patients with heterogeneous tumor size and locations, the benefits of combined therapy appeared to be substantial in three cases. Four of the 8 patients remain alive, 3 in tumor remission with a current median follow-up of 12 months. This favorable outcome is possibly related to adequate cisplatin perfusion of the margin tumor regions 24 h after systemic administration of chemotherapy combined with LITT [8,11,14]. A total of 4 patients expired after combined laser and chemotherapy (survival range of 4–8 months). Three had disease progression and one died of intercurrent illness while in tumor remission. This pilot clinical study indicates that combining laser ablation and chemotherapy may become an effective method providing palliative treatment for some patients with advanced head and neck tumors. However, longer follow-up in a larger cohort of patients and randomized clinical trials are needed to fully validate LITT and chemotherapy as a less invasive combined treatment option for palliation of head and neck cancer.

### *Acknowledgements*

The authors are thankful for Mrs. Portia Gecewicz and Mrs. Neva Jongewaard for their technical assistance and continuing support for our research program. This study was supported by the Division of Head and Neck Surgery, and the Jonsson Comprehensive Cancer Center, UCLA School of Medicine, and NIH Grant # CA65053-O1R.

### *References*

[1] Joffee, S.N., Tajiri, H., Oguro, Y. *et al.* Laserthermia: A new method of interstitial local hyperthermia using the contact Nd : YAG laser. *Radiol. Clinic North Am.* 1989; **27**: 611–620.

[2] Feyh, J., Gutmann, R., Leunig, A. *et al.* MRI-Guided Laser Interstitial Thermal Therapy (LITT) of head and neck tumors: progress with a new method. *J. Clin. Laser Med. Surg.* 1996; **14**: 361–366.

[3] Sturesson, C. and Andersson-Engels, S. Theoretical analysis of transurethral laser induced thermo-therapy for treatment of benign prostatic hyperplasia. *Phys. Med. Biol.* 1996; **41**: 445–463.

[4] Schroder, T., Castren-Persons, M., Lehtinen, A. *et al.* Percutaneous interstitial laser hyperthermia in clinical use. *Ann. Chir. Gynaecol.* 1994; **83**: 286–290.

[5] Saxton, R.E., Paiva, M.B., Lufkin, R.B. *et al.* Laser photochemotherapy: A less invasive approach for treatment of cancer. *Semin. Surg. Oncol.* 1995; **11**: 283–289.

[6] Semler, P., Koch, K. and Schumacher, W. Eine neue Möglichkeit zur Behandlung stenosierender Tumoren im oberen Gastrointestinaltrakt. *Dtsch. Med. Wochenschr.* 1985; **100**: 1731–1732.

[7] Mache, H.N., Koch, K., Stadler, D. *et al.* The therapeutic possibilities of the combination of laser resection and the afterloading technique as seen by the endoscopist. *Tumor Diag. U. Therap.* 1986; **7**: 5–9.

[8] Firusian, N. Chemo- and laser therapy of advanced tumors of the upper gastrointestinal tract. *Onkologie* 1988; **11**(Suppl. 2): 23–33.

[9] Zenone, T., Romestaing, P., Lambert, R. *et al.* Curative non-surgical combined treatment of the oesophagus. *Eur. J. Cancer* 1992; **28**: 1380–1386.

[10] Highley, M.S., Parnis, F.X., Trotter, G.A. *et al.* Combination chemotherapy with epirubicin, cisplatin and 5-fluorouracil for the palliation of advanced gastric and oesophageal adenocarcinoma. *Br. J. Surg.* 1994; **81**: 1763–1765.

[11] Mason, R. Palliation of malignant dysphagia: An alternative to surgery. *An. R Coll. Surg. Engl.* 1996; **78**: 457–462.

[12] Lueder, G.T. and Goyal, R. Visual function after laser hyperthermia and chemotherapy for macular retinoblastoma. *Am. J. Opthalmol.* 1996; **121**: 582–584.

[13] Gallie, B.L., Budning, A., DeBoer, G. *et al.* Chemotherapy with focal therapy can cure intraocular retinoblastoma without radiotherapy. *Arch. Opthalmol.* 1996; **114**: 1321–1327.

[14] Murphree, A.L., Villablanca, J.G., Deegan, W.F. *et al.* Chemotherapy plus local treatment in the management of intraocular retinoblastoma. *Arch. Opthalmol.* 1996; **114**: 1348–1356.

[15] Paiva, M.B., Blackwell, K.E., Saxton, R.E. *et al.* Palliative laser therapy for recurrent head and neck cancer: A Phase II clinical study. *Laryngoscope* 1998; **108**: 1277–1283.

[16] Hettinga, J.V., Lemstra, W., Meijer, C. *et al.* Hyperthermic potentiation of cisplatin toxicity in a human small cell lung carcinoma cell line and a cisplatin resistant subline. *Int. J. Hyperthermia* 1994; **10**: 795–805.

[17] Arancia, G., Crateri Trovalusci, P., Mariutti, G. *et al.* Ultrastructural changes induced by hyperthermia in Chinese hamster V79 fibroblasts. *Int. J. Hyperthermia* 1989; **5**: 341–350.

[18] Zanke, B.W., Boudreau, K., Rubie, E. *et al.* The stress-activated protein kinase pathway mediates cell death following injury induced by cis-platinum, UV irradiation or heat. *Curr. Biol.* 1996; **6**: 606–613.

[19] Ohno, S., Siddik, Z.H., Kido, Y. *et al.* Thermal enhancement of drug uptake and DNA adducts as a possible mechanism for the effect of sequencing hyperthermia on cisplatin-induced cytotoxicity in L1210 cells. *Cancer Chemother. Pharmacol.* 1994; **34**: 302–306.

[20] Beketic-Oreskovic, L., Jaksic, M., Oreskovic, S. *et al.* Hyperthermic modulation of resistance to cis-diammine-dichloroplatinum(II) in human larynx carcinoma cells. *Int. J. Hyperthermia* 1997; **13**: 205–214.

[21] Hettinga, J.V., Lemstra, W., Meijer, C. *et al.* Heat-shock protein expression in cisplatin-sensitive and-resistant human tumor cells. *Int. J. Cancer* 1996; **67**: 800–807.
[22] Los, G., van Vugt, M.J., den Engelse, L. *et al.* Effects of temperature on the interaction of cisplatin and carboplatin with cellular DNA. *Biochem. Pharmacol.* 1993; **46**: 1229–1237.
[23] Rauko, P., Novotny, L. and Balazova, E. DNA breakage and inactivation resulting from hydroxylamine and/or cis-diamminedichloroplatinum(II) interactions with plasmid DNA. *Int. J. Bioch.* 1993; **25**: 1475–1481.
[24] Burris, H.A., Vogel, C.L., Castro, D.J. *et al.* Intratumoral Cisplatin/epinephrine injectable gel as a palliative treatment for accessible solid tumors: A multicenter pilot study. *Otolaryngol. Head Neck Surg.* 1998; **118**: 496–503.
[25] Paiva, M.B., Saxton, R.E., VanderWerf, Q.M. *et al.* Cisplatinum and interstitial laser therapy for advanced head and neck cancer: A preclinical study. *Laser Surg. Med.* 1997; **21**: 423–431.
[26] Paiva, M.B., Graeber, I.P., Castro, D.J. *et al.* Laser and cis-platinum for treatment of human squamous cell carcinoma. *Laryngoscope* 1998; **108**: 1269–1278.

